# A hierarchical brain MRI atlas of the coppery titi monkey (*Plecturocebus cupreus*)

**DOI:** 10.1016/j.neuroimage.2026.121921

**Published:** 2026-04-10

**Authors:** Alita Jesal D Almeida, Brad A. Hobson, Anelise Caceres, Sarah Tam, John P. Paulus, Claudia Manca, Anand A. Joshi, Sara M. Freeman, Karen L. Bales, Abhijit J. Chaudhari

**Affiliations:** a Department of Biomedical Engineering, University of California-Davis College of Engineering, Davis, CA, 95616, USA; b Department of Radiology, University of California-Davis School of Medicine, Sacramento, CA, 95817, USA; c Center for Molecular and Genomic Imaging, University of California-Davis, Davis, CA, 95616, USA; d Department of Neuroscience, University of California-Davis Center for Neuroscience, Davis, CA 95616, USA; e Department of Psychology, University of California-Davis College of Letters and Science, Davis, CA 95616, USA; f Ming Hsieh Department of Electrical and Computer Engineering, University of Southern California, Los Angeles, CA 90089-2560, USA; g Department of Biology, Utah State University, Logan, UT 84322, USA; h California National Primate Research Center, Davis, CA 95616, USA

**Keywords:** Titi monkey, Hierarchical brain atlas, Population template, Social bonding, [^11^C]GR103545 PET

## Abstract

**Introduction:**

The coppery titi monkey (*Plecturocebus cupreus*) is an essential nonhuman primate model for social neuroscience, yet neuroimaging studies have been severely constrained by the paucity of standardized atlases. We address this gap by introducing the first MRI-based atlas package for the titi monkey brain that includes a single-subject atlas (UC Davis Titi monkey Neuroimaging Atlas (UCD-TiNA)), alongside a population atlas (UCD-TiNA_group) and a manually-segmented atlas compilation (UCD-TiNA_mac).

**Methods:**

UCD-TiNA comprises 74 hierarchically organized regions delineated from the MRI of a representative adult brain, along with whole brain, gray and white matter masks. These segmentations were propagated to the population template (UCD-TiNA_group, N = 17 monkeys) generated to enhance generalizability. A manually-segmented atlas compilation (UCD-TiNA_mac, N = 6 monkeys, 14 regions) was created to enable multi-atlas segmentation approaches. UCD-TiNA and UCD-TiNA_mac were evaluated in a [^11^C]GR103545 PET and MRI study (N = 42 monkeys) to quantify regional kappa opioid binding.

**Results:**

The warped UCD-TiNA achieved a high concordance with the manually-segmented UCD-TiNA_mac labels (median Dice = 0.74). Regional [^11^C]GR103545 binding potential was consistent with published patterns. Quantitative PET analyses showed <1% median error and a high correlation (Spearman r = 0.99) between the warped and manually-segmented labels.

**Discussion:**

This work delivers the first *in vivo* atlas package to enable standardized, reproducible and cross-modal analyses of titi monkey neuroimaging data. By providing a common anatomical reference, this atlas package should facilitate rigorous and harmonized data processing, supporting high-throughput and longitudinal investigations in social neurobiology and informing translational research on social behavior.

## 1. Introduction

Understanding the neural circuitry underlying human social attachment is critical for advancing our mechanistic understanding of interpersonal behavior and developing tools and interventions for monitoring and treating disorders of social affiliation. A well-characterized form of social attachment, the pair bond, consists of physiological and behavioral traits observed between two sexually mature individuals that extend beyond conventional animal mating patterns, which are transactional in nature (Young and Wang, 2004, Bales et al., 2021). Brain regions and brain networks associated with pair bonds in humans have been identified through functional MRI (fMRI) (Fisher et al., 2005, Blumenthal and Young, 2023, Bartels and Zeki, 2004, Bartels and Zeki, 2000). These studies highlight the activation of reward circuits in brain regions rich in oxytocin and/or vasopressin during interactions involving a pair-bonded partner (Insel and Young, 2001). Associations between differences in brain structures, such as gray matter volume and attachment styles (measured with voxel-based morphometry (Zhang et al., 2018, Leblanc et al., 2017, Acosta et al., 2018, Benetti et al., 2010)) as well as differences in white matter connectivity observed in bonds between infants and their mothers (Serra et al., 2015, Dégeilh et al., 2023) have also been established. While the utilization of PET to investigate pair bonded behavior in humans is fairly limited (Takahashi et al., 2015), it has been used to study addiction (Martinez et al., 2019, Domi et al., 2018, Rorick-Kehn et al., 2014, Mello and Negus, 2006), mood (Miller et al., 2018, Lalanne et al., 2014, Yatham et al., 2000, Su et al., 2014) and eating (Gianni et al., 2020, Frank and Kaye, 2005) disorders. Although human imaging studies offer valuable insights into the neurobiological mechanisms underlying social bonding, experimental investigations are inherently constrained due to the limited capacity to obtain longitudinal data under tightly controlled conditions, restricted access to neural tissue, and the inability to perform invasive validation studies. Animal models with neurobiological and behavioral homology to humans offer a translational bridge to mechanistically interrogate social circuits using techniques and paradigms unavailable in human research (Toth and Neumann, 2013, Blanchard et al., 2001); however, animal models with extensive neuroimaging toolkits such mice, rats, and macaques do not form pair bonds.

There are limited mammals that display pair bonding behavior, among these, prairie voles, a rodent model of pair bonding, are the most widely studied (Winslow et al., 1993, Walum and Young, 2018, Insel and Shapiro, 1992). However, the translatability of this species is limited due to the substantial differences of their neuroendocrine system compared to humans and other primates (Freeman and Young, 2016), indicative of altered mechanisms of action (Freeman et al., 2014). By comparison, nonhuman primates (NHPs) share profound neurobiological, psychological, social, and developmental homologies with humans due to evolutionary proximity, making them invaluable models for elucidating mechanisms underlying complex human behavior and cognition (Friedman et al., 2017, Novak and Suomi, 1991, Capitanio and Emborg, 2008). In particular, the coppery titi monkey (*Plecturocebus cupreus*) is a South American NHP species that forms pair bonds and thus offers a more generalizable and superior translational animal model due to its neurological similarities to humans (Bales et al., 2017, Hinde et al., 2016, Ragen et al., 2012). Unlike polygamous NHPs (French et al., 2018), titi monkeys naturally exhibit monogamous pairing, partner preference, separation distress, and stress-buffering effects of the partner’s presence, the key components of human pair bonding (Carp et al., 2016, Savidge and Bales, 2020, Escriche Chova et al., 2023). Moreover, the recently published genomic data for the titi monkey (Pfeifer et al., 2024) further establishes it as a robust model for advancing investigations into mechanisms that govern social bonding and social separation between pair-bonded individuals.

Imaging studies across social experimental conditions have been conducted in titi monkeys using PET (Razo et al., 2022, Maninger et al., 2017, Bales et al., 2007, Hostetler et al., 2017, Almeida et al., 2024), providing an *in vivo* assessment and enabling longitudinal studies within the same animal in a controlled setting. These studies have investigated molecular processes such as glucose metabolism (Razo et al., 2022, Maninger et al., 2017, Bales et al., 2007), dopamine (Hostetler et al., 2017) and kappa opioid receptor (KOR) (Almeida et al., 2024) activity, which when correlated with behavioral outcomes (Razo et al., 2022), facilitate a more comprehensive understanding of the neurobiological mechanisms underlying the expression pair bonding traits in this monkey model. However, due to the lack of standardized anatomical atlases and image analysis resources tailored to the titi monkey brain, these studies have relied on time consuming and labor-intensive manual segmentations, limiting analyses to a small number of anatomical regions per study, introducing operator bias (Harvey, 2021, Koo and Li, 2016), hindering longitudinal studies and precluding large-scale or multisite harmonization. The establishment of standardized atlas-based approaches specific to titi monkeys is therefore essential to harness the translational potential of this model.

Atlas-based segmentation of MRI data is routine in human (Evans et al., 2012, Despotović et al., 2015, Cabezas et al., 2011) and laboratory animal studies (Kovačević et al., 2005, Chuang et al., 2011, Schweinhardt et al., 2003, Schwarz et al., 2006, Newman et al., 2009, Liu et al., 2018, Jung et al., 2021, Saleem et al., 2021, Weiss et al., 2021, Hartig et al., 2021, Reveley et al., 2016), enabling reproducible, semi-automated analyses at scale. While an atlas can be generated from a single subject, population-based group templates (Seidlitz et al., 2018), created by linear or non-linear averaging of MR scans across multiple subjects (McLaren et al., 2009, Fonov et al., 2011), provide a more representative prior that can improve segmentation accuracy over single-subject templates (Yang et al., 2020). Furthermore, multi-atlas segmentation methods leveraging multiple manually-segmented reference atlases, where labels from these atlases are warped to individual subject scans and fused using voting schemes (Polikar, 2006). They may improve segmentation accuracy by reducing the bias inherent in single-atlas methods (Lötjönen et al., 2010, Huo et al., Wang et al., 2013, Bai et al., 2013). Critically, establishing reference datasets of manual segmentation enables rigorous validation of automated warping algorithms and provides benchmarking standards for algorithm development and refinement. Such comprehensive tools, combining hierarchical atlases, population-based templates, and multi-atlas compilations are well established for humans and are available for other species, including rhesus macaques (*Macaca mulatta*), but remain absent for titi monkeys.

To develop a standardized neuroimaging workflow for titi monkeys, we first established a standardized anterior commissure-posterior commissure (AC-PC) reference space for the brain. This stereotaxic framework, analogous to that developed by Talairach and Tournoux (Talairach) used in human neuroimaging, was adapted. The integrated atlas package was developed with three complementary components:

1. University of California Davis - Titi monkey Neuroimaging Atlas (UCD-TiNA), a hierarchical 74 volumes of interest (VOI) brain MRI anatomical atlas, created from a single subject with three levels of cortical and sub-cortical classifications;
2. UCD-TiNA_group, a population-based brain template, derived from MRI scans of 17 monkeys incorporating the same 74 VOIs as UCD-TiNA, to enhance registration generalizability; and
3. UCD-TiNA_mac, a manually-segmented MRI atlas compilation of 14 social-bonding-associated VOIs from 6 monkeys, designed as a reference to benchmark warping accuracy and enable multi-atlas fusion approaches.

We additionally generated whole brain, white matter, and gray matter masks for UCD-TiNA and UCD-TiNA_group to enable streamlined downstream analysis.

To rigorously evaluate atlas validity and demonstrate its utility, we warped these atlases via co-registered MRI to perform regional analysis of PET scans with the radiotracer [^11^C]GR103545 that selectively binds to the KOR (Almeida et al., 2024), in an independent cohort of N = 42 titi monkeys, systematically comparing warped atlas-based segmentations to manual segmentation.

## 2. Methods

### 2.1. Animal selection and housing

All animal experiments were conducted in AAALAC-accredited facilities with approval from the University of California, Davis Institutional Animal Care and Use Committee (IACUC; protocol number: 23,483). Adult titi monkeys at the California National Primate Research Center were socially housed in cages under a 12:12 light:dark cycle with temperature maintained at 21 °C. They were fed a standard diet of monkey chow, banana, rice, cereal, apple, and carrot twice a day.

For the primary atlas (UCD-TiNA), an adult female titi monkey (1.84 years old, weighing 1.26 kg) was selected, with images meeting standard quality assurance criteria, such as no history of brain disorders or neuropathology, absence of obvious motion artifacts or structural abnormalities in MRI, brain volume closest to the cohort median, and bilateral symmetry. This representative individual was identified upon systematically parsing through our imaging database. Although longitudinal data on brain development and aging in titi monkeys are not currently available, extrapolation from our own cross-sectional analyses in titi monkey brains and comparison to published developmental studies in rhesus macaques (Kim et al., 2020) suggest the subject’s age corresponds to a young adult stage attaining a near-plateau phase of brain maturation, with subsequent morphological changes expected to be modest during adulthood. For UCD-TiNA_group (N = 17), we included adult monkeys (10 males and 7 females, ages 5.09 ± 1.81 years, weighing 1.23 ± 0.16 kg) selected at random within the age window of 1.5 – 9.0 years to mitigate age-related atrophy. For UCD-TiNA_mac (N = 6), adult animals (4 males and 2 females, 9.44 ± 3.87 years old, weighing 1.37 ± 0.09 kg) were selected at random to capture anatomical variability and included 4 animals of typical adult age and morphology, plus 2 geriatric animals (ages 10.59 and 16.85 years) representing morphological extremes. Lastly, the validation PET cohort (N = 42; 21 males and 21 females, 8.29 ± 3.27 years old, weighing 1.27 ± 0.15 kg) was an independent group.

### 2.2. MRI acquisition

T_1_-weighted anatomical MR images were acquired using a 1.5T GE Signa LX9.1 scanner (General Electric Corporation, Milwaukee, WI) equipped with a 7.62 cm (3.00 in) surface coil. Imaging was performed in the coronal plane using a 3D spoiled gradient echo (SPGR) pulse sequence with the following parameters: echo time (TE) = 7.9 ms, repetition time (TR) = 22.0 ms, flip angle = 30°, field of view = 8 cm, number of excitations = 3, and matrix size = 256 × 256, resulting in an in-plane voxel resolution of 0.3125 × 0.3125 mm^2^ and a slice thickness of 1 mm.

### 2.3. Post-processing of MRI data

Raw MR images were skull-stripped using BrainSuite (v23a) (Shattuck and Leahy, 2002), with manual correction. MRIs were then bias corrected using N4ITK (Tustison et al., 2010) and were interpolated along the z axis to obtain an isotropic voxel size (0.33 mm^3^) to enhance warping algorithm convergence.

### 2.4. Construction of the primary atlas (UCD-TiNA)

**Fig. 1.**
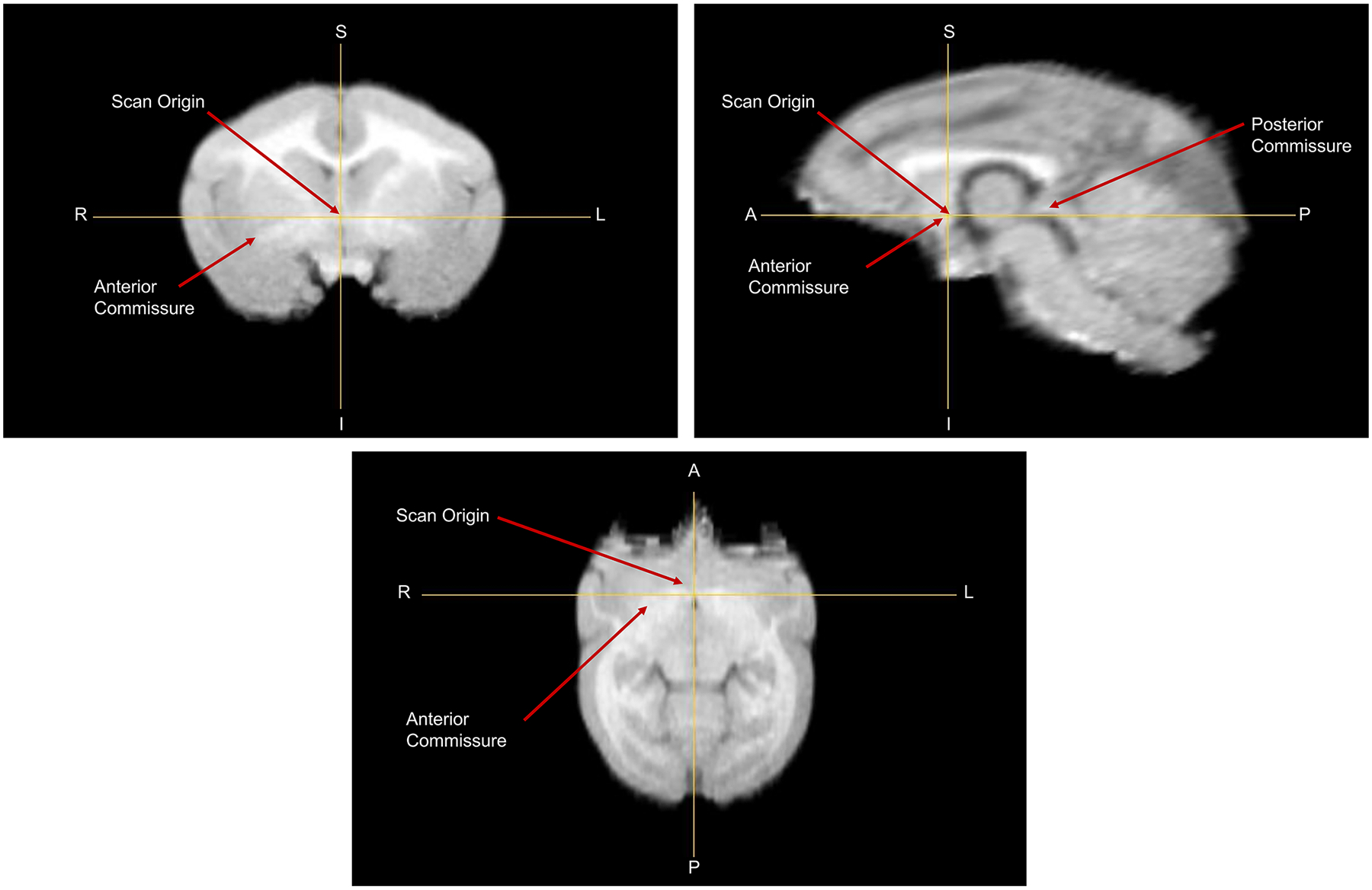
The MRI selected to develop UCD-TiNA was first manually mapped to AC-PC space. The horizontal plane was assigned to be the line connecting the dorsal apex of the anterior commissure to the ventral nadir of the posterior commissure. The apex of the anterior commissure was set to be the origin of the scan.

The MRI scan selected for UCD-TiNA was aligned to standard neuroanatomical orientation, the AC-PC space. The dorsal apex of the anterior commissure and the ventral nadir of the posterior commissure were identified on coronal slices, and a line connecting them (Fig. 1) defined the horizontal axis with the dorsal apex of the anterior commissure demarcated as the origin. All animals in the study were normalized to this titi monkey reference space via rigid registration.

Manual segmentation of the atlas was performed using Slicer 3D, an open-source image analysis software (Fedorov et al., 2012). The Cortical Hierarchy Atlas of the Rhesus Macaque (CHARM) (Jung et al., 2021), the Subcortical Hierarchy Atlas of the Rhesus Macaque (SARM) (Hartig et al., 2021) and data from prior histological and autoradiographical studies in titi monkeys (Freeman et al., 2014; Ragen et al., 2015; University of Wisconson-Madison Brain Collection) were utilized as references for segmentation (details are provided in Supplementary Figs. 1–3). The CHARM and SARM, population-based, hierarchical brain atlases of the rhesus macaque, generated through iterative nonlinear alignment of 31 rhesus monkeys (25 males and 6 females), provided high resolution, multi-level anatomical-based parcellations of a non-human primate brain closer in scale and cortical complexity to titi monkeys than similar quality atlases in humans.

Given the titi monkey’s smoother cortical surface (fewer sulci than rhesus macaques), direct transfer of rhesus parcellations was not feasible. Therefore, comparative neuroanatomical methods were employed, wherein, in the absence of deep sulci, cortical VOI boundaries were defined using shallow sulcal indentations present on the titi cortical surface. Examining the skull-stripped 3D renderings of both brains, the 4 primary brain lobes were defined (Supplementary Fig. 2). Structures within each lobe on coronal slices were further delineated based on gray-white matter contrast across slices. This systematic approach ensured consistent, graded VOI transitions across slices rather than sharp discontinuities and abrupt VOI transitions. VOIs were selected across the five levels of CHARM based on their visibility and contrast in UCD-TiNA. Where the smoother titi cortex did not support finer parcellation, cortical VOIs were merged or omitted (Supplementary Fig. 3). Sub-cortical VOIs were predominantly constrained to Levels 2 and 3 of SARM due to the scan resolution of our dataset and the slight blurring introduced by the interpolation of slices. Whenever possible, initial regional delineations were cross-referenced and adjusted using available acetylcholinesterase staining and autoradiography data for oxytocin, vasopressin 1a, and opioid receptor distributions (Freeman et al., 2014; Ragen et al., 2015).

Regional delineations in the atlas were broadly categorized into white and gray matter structures. The white matter tract consisted of a single sub-hierarchical delineation, the centrum semiovale, which was included due to its increased utilization as a reference region for PET reference tissue kinetic modeling (Koole et al., 2019; Lyoo et al., 2015; Li et al., 2022; Rossano et al., 2020). The gray matter delineations were primarily divided into cortical and subcortical structures; each further organized into three levels of hierarchy.

Cortical VOIs were arranged hierarchically: Level 1 comprised 4 lobes (frontal, parietal, temporal, occipital); Level 2 further divided these into 15 VOIs; Level 3 consisted of 24 VOIs with 7 from Level 2 subdivided into 16 additional VOIs, while 8 remained undivided. Bilateral symmetry was not assumed, and left/right hemispheres were segmented separately to avoid errors from asymmetric anatomy.

Subcortical structures were organized similarly: Level 1 comprised 4 major divisions (telencephalon, diencephalon, mesencephalon, metencephalon); Level 2 yielded 14 VOIs (11 unilateral); Level 3 further subdivided 7 of these into 21 VOIs (10 unilateral), yielding 28 subcortical VOIs in total. The full hierarchy is detailed in Table 1.

The drawings were performed by a researcher (with four years of experience analyzing titi monkey brain image data and five years of experience in preclinical imaging) who was trained by an expert in preclinical neuroanatomy (with six years of experience analyzing titi monkey brain image data and 14 years of experience in brain imaging), with iterative feedback. A senior expert (~20 years of experience with titi monkey brain imaging) reviewed and refined all segmentations to ensure anatomical accuracy. The VOI delineations and the whole brain, white and gray matter masks for UCD-TiNA can be found on our Github page (https://github.com/ajchaudhari/UCD-TiNA/tree/main/UCD-TiNA).

To assess intra- and inter-rater variability, the primary segmenter re-segmented UCD-TiNA across a subset of Level 3 VOIs eight months after the initial drawings and these same regions were additionally, independently segmented by a senior expert.

### 2.5. Generation of the group atlas template (UCD-TiNA_group)

**Fig. 2.**
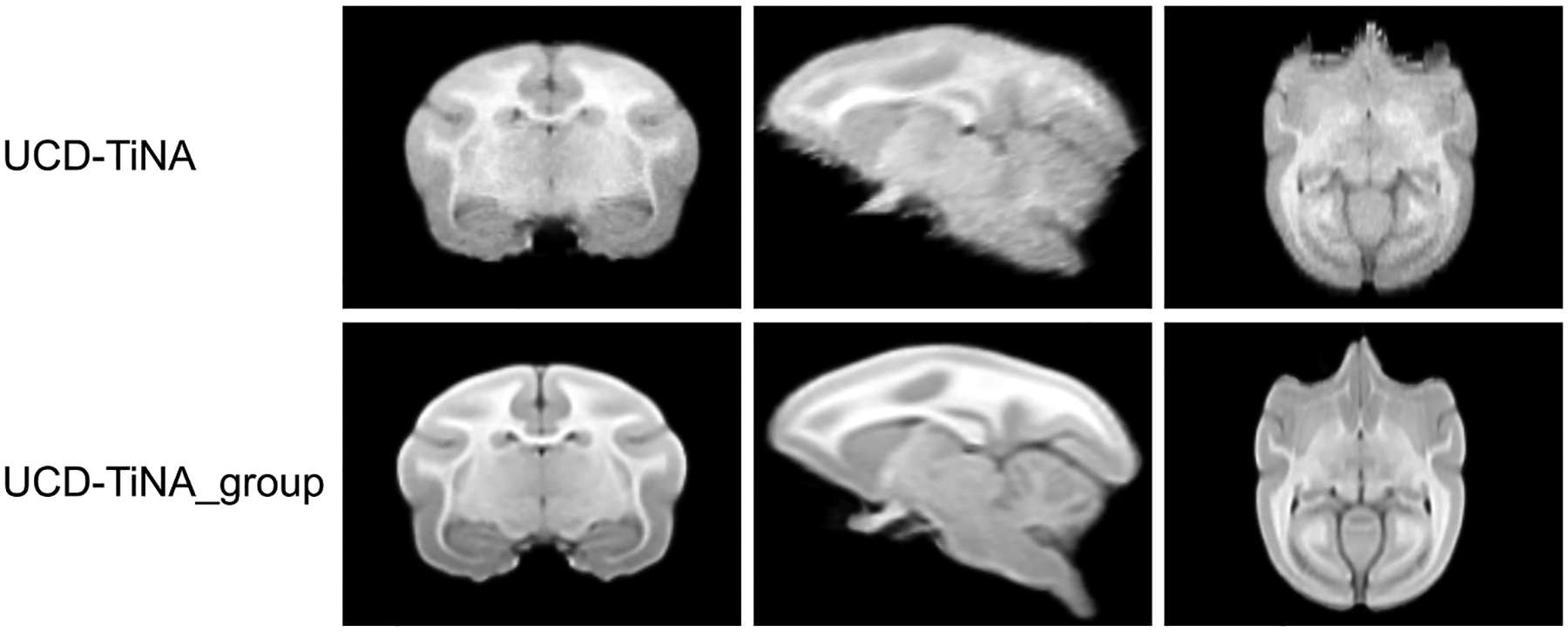
Representative slices from UCD-TiNA (row 1) showing coronal (left), sagittal (middle) and axial (right) views, and corresponding slices from the population-based group brain template, UCD-TiNA_group, generated using *antsMultivariateTemplateConstruction2.sh* (row 2). Note that the presence of the brainstem in UCD-TiNA_group can be attributed to a larger field of view present in some scans within the dataset.

UCD-TiNA_group, was constructed from N = 17 individual MRIs, each aligned to AC-PC reference space via rigid registration to UCD-TiNA. Population-based averaging employed the Advanced Normalization Tools Suite (ANTs)’s *antsMultivariateTemplateConstruction2* script (Avants et al., 2011) (Fig. 2), with the ANTs SyN nonlinear registration (cross-correlation similarity metric) (Avants et al., 2014; Avants et al., 2009). The process required 9 h and 28 min. Corresponding VOI delineations and whole brain, white and gray matter masks from UCD-TiNA were then propagated to the population template. The UCD-TiNA_group template, the whole brain, white and gray matter masks and VOI delineations can be accessed through our Github page (https://github.com/ajchaudhari/UCD-TiNA/tree/main/UCD-TiNA_group).

### 2.6. Processing and development of the manually-segmented atlas compilation (UCD-TiNA_mac)

UCD-TiNA_mac consists of manual segmentations from N = 6 animals, and contains segmentations of 14 VOIs, primarily implicated in social bonding. The 6 bilateral cortical VOIs included the dorsolateral prefrontal cortex, somatosensory cortex, rhinal cortex, inferior temporal cortex, superior temporal region and the insula. The subcortical VOIs included 4 bilateral VOIs – the amygdala, caudate, putamen and nucleus accumbens and 4 unilateral VOIs – the lateral septum, hypothalamus, ventral and medial thalamus. Each VOI was manually delineated using UCD-TiNA as the anatomical reference, with expert review and refinement to ensure consistency and accuracy. The VOIs selected belong to Level 3 of UCD-TiNA. Regional labels for the 6 animals and their corresponding MRIs can also be accessed via our Github page (https://github.com/ajchaudhari/UCD-TiNA/tree/main/UCD-TiNA_mac).

### 2.7. PET and MRI acquisition for the evaluation data set

The PET radiotracer [^11^C]GR103545 synthesis was performed as described previously (Nabulsi et al., 2011) with a mean molar activity of 140.22 ± 26.0 PBq/mol at the end of synthesis. Titi monkeys were anesthetized with 1–2% isoflurane and positioned head-first on a custom bed of a dedicated primate brain PET scanner (PiPET, Brain Biosciences, Rockville, MD). The dynamic 110 min PET acquisition began approximately 15 s prior to injection of [^11^C]GR103545 (injected activity 52.93 ± 8.63 MBq, injected mass 0.28 ± 0.04 μgm/kg). Data were reconstructed with an isotropic voxel size of 0.8 mm^3^ using framing of 6 × 10 s, 8 × 30 s, 5 × 60 s, 4 × 300 s, 8 × 600 s. A single-pass “cold” transmission scan was acquired prior to the injection for attenuation and scatter correction. MRIs were acquired for each monkey at the same time-point using the same acquisition protocol as for atlas creation.

### 2.8. Dataset postprocessing and analysis

The PET data were motion-corrected via rigid registration to a reference frame and co-registered to the MRIs on PMOD (v4.4, PMOD Technologies, Zürich, Switzerland). To generate regional labels, UCD-TiNA was warped to each subject’s MRI using ANTs B-Spline SyN with a step size of 26 (Avants et al., 2014; Avants et al., 2008). The warping parameters were selected based on a separate optimization to evaluate the impact of varying the transform type, similarity metrics, gradient step size, spline distance, shrink and convergence factors, weight of the regularization term and histogram matching on the warping performance (Supplementary Table 2). These settings were empirically tuned to our 1.5T titi monkey data to balance registration accuracy and anatomical plausibility; a comprehensive comparison of alternative ANTs parameterizations for small non-human primate brains will be presented in a separate methodological report.

Warping accuracy was assessed by comparing manually-segmented the 14 UCD-TiNA_mac VOIs to ANTs-warped UCD-TiNA VOIs using: Dice Coefficient (DC) (Zou et al., 2004), a metric of overlap, and the Hausdorff Distance (HD) (Huttenlocher et al., 1993), a metric to determine the extent of deviation from the region’s boundary.

### 2.9. PET kinetic modeling

Regional non-displaceable binding potential (BP_ND_) was calculated utilizing the Simplified Reference Tissue Model (SRTM) (Lammertsma and Hume, 1996), with the cerebellum as the reference region in PMOD (v4.4). Analysis focused on the 14 UCD-TiNA_mac brain VOIs that are relevant to social bonding. Additional details on PET and MRI data analysis are available in our previous work (Almeida et al., 2024). Regional BP_ND_ estimates were compared between manual and warped segmentations for the 14 UCD-TiNA_mac VOIs using Spearman correlation, Bland-Altman analysis and root mean squared error (RMSE).

## 3. Results

### 3.1. UCD-TiNA hierarchical segmentation

**Fig. 3.**
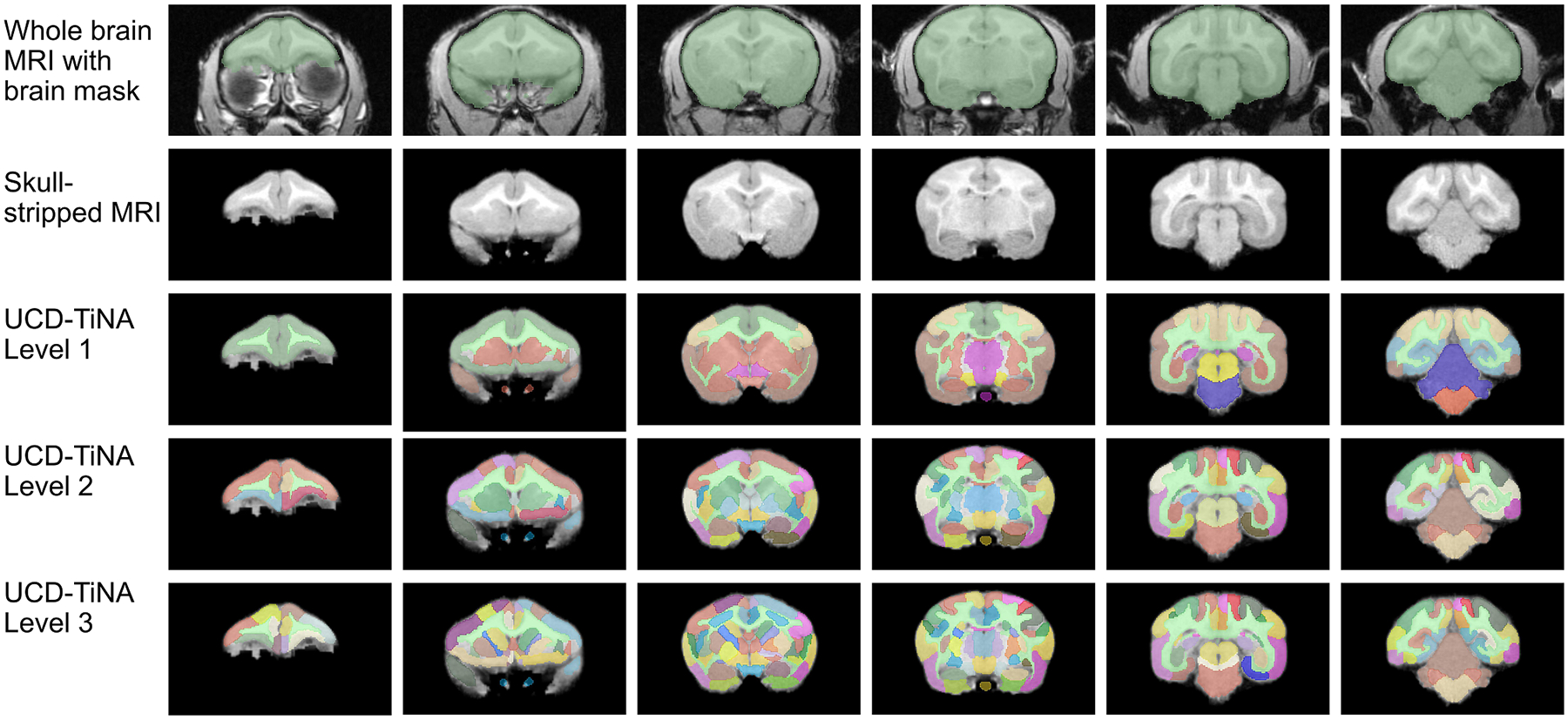
Representative coronal slices showing UCD-TiNA delineations across slices the brain. Row 1 presents the brain MRI with the whole brain mask (green) overlaid on it. Row 2 shows the skull-stripped MRI. Rows 3, 4 and 5 illustrate the hierarchical brain segmentations corresponding to Levels 1, 2 and 3 of UCD-TiNA, respectively (in color) overlaid on the skull-stripped MRI.

**Fig. 4.**
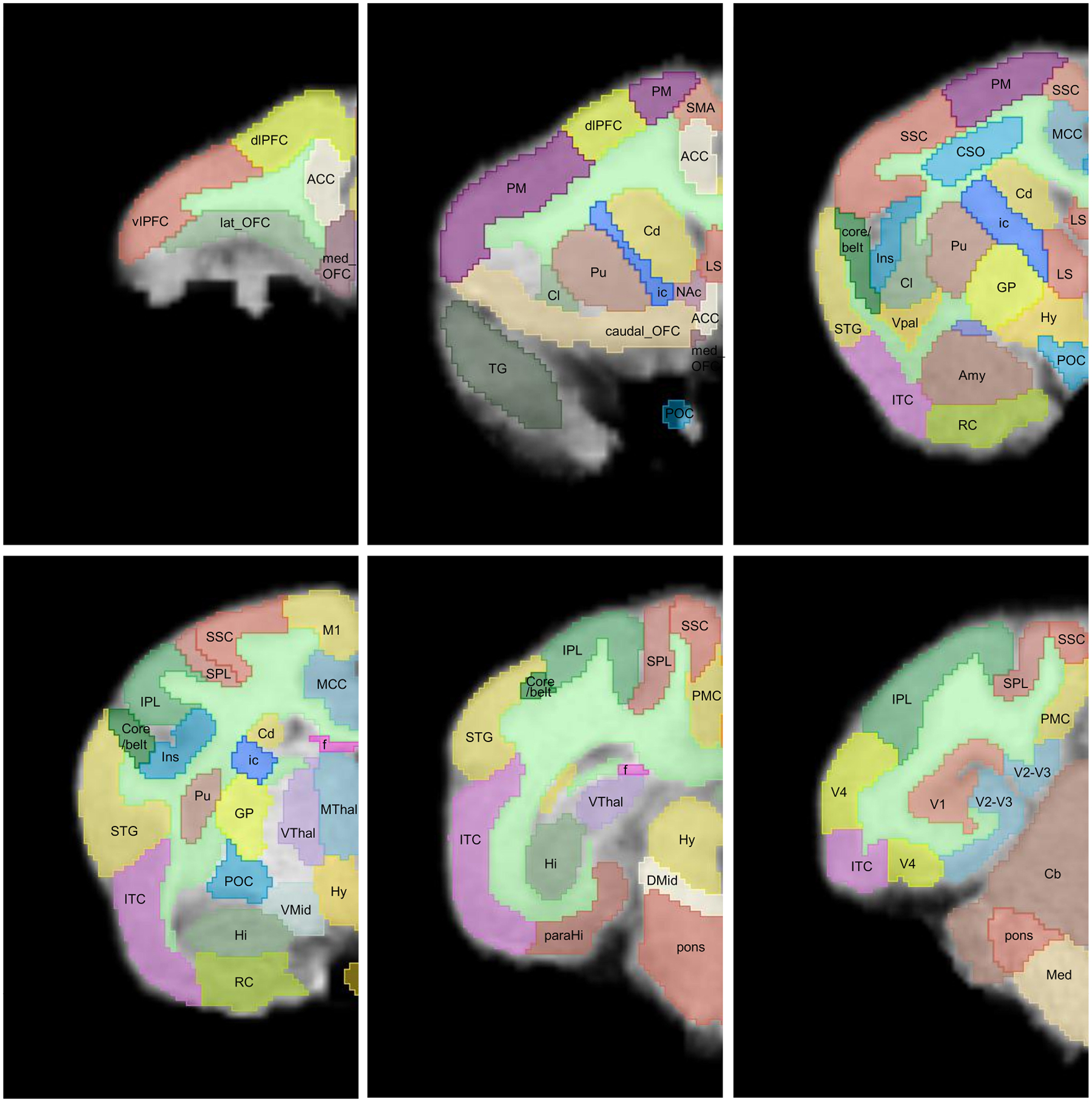
Coronal slices illustrating hierarchical Level 3 VOIs (color) overlaid on skull-stripped MRI slices of UCD-TiNA. Abbreviations are as follows: Anterior Cingulate Cortex: ACC, Mid Cingulate Cortex: MCC, Medial Orbitofrontal Cortex: med_OFC, Lateral Orbitofrontal Cortex: lat_OFC, Caudal Orbitofrontal Cortex: caudal_OFC, Dorsolateral Prefrontal Cortex: dlPFC, Ventrolateral Prefrontal Cortex: vlPFC, Medial Supplementary Motor Areas: SMA, Premotor Cortex: PM, Primary Motor Cortex: M1, Somatosensory Cortex: SSC, Superior Parietal Lobule: SPL, Inferior Parietal Lobule: IPL, Posterior Medial Cortex: PMC, Parahippocampal Cortex: ParaHi, Rhinal Cortex: RC, Temporal Pole: TG, Inferior Temporal Cortex: ITC, Superior Temporal Region: STG, Core and Belt Areas of Auditory Cortex: core/belt, Insula: Ins, Primary Visual Cortex: V1, Extrastriate Visual Areas: V2-V4, Claustrum: Cl, Ventral Pallium: Vpal, Amygdala: Amy, Preoptic Complex: POC, Caudate: Cd, Putamen: Pu, Internal Capsule: ic, Nucleus Accumbens: NAc, Hypothalamus: Hy, Epithalamus: EpiThal, Ventral Thalamus: Vthal, Medial Thalamus: Mthal, Dorsal Midbrain: Dmid, Pons: pons, Cerebellum: Cb, Medulla: Med, Centrum Semiovale: CSO.

UCD-TiNA comprises 74 VOIs spanning three hierarchical levels across cortical and subcortical gray matter, plus white matter. Fig. 3 shows the full hierarchical architecture, while Fig. 4 illustrates labeled slices from hierarchical Level 3, both of which are on coronal slices progressing through the brain. White matter delineations were omitted in subcortical regions where visual inspection by two collaborating observers were unable to reach consensus due to partial voluming. For tracts surrounding cortical regions, CHARM (Jung et al., 2021) and histological sections of the titi monkey brain (University of Wisconson-Madison Brain Collection) served as a reference for defining their trajectories (Supplementary Fig. 4). An assessment of signal-to-noise ratio (SNR) was performed to evaluate the quality of the structural data. SNR values of 31.14 (gray matter) and 33.48 (centrum semiovale) were obtained by dividing mean signal intensity by the standard deviation within each VOI. These SNR values were comparable in magnitude to those reported for 1.5T structural brain MRI in multi-center human studies (Magnotta et al., 2006).

At the finest resolution (Level 3), the atlas consists of 52 VOIs spanning diverse anatomical scales. VOI volumes ranged substantially: from small nuclei such as the ventral pallidum (0.005 cm^3^) to large structures including the cerebellum (1.57 cm^3^) and cortical lobes (e.g., occipital cortex: 1.23 cm^3^). Complete VOI characteristics including voxel counts, volumes, and surface areas are listed in Table 2. VOI surface area and volumes were computed using a smoothed, closed surface model of the segmentation (Fedorov et al., 2012).

**Fig. 5.**
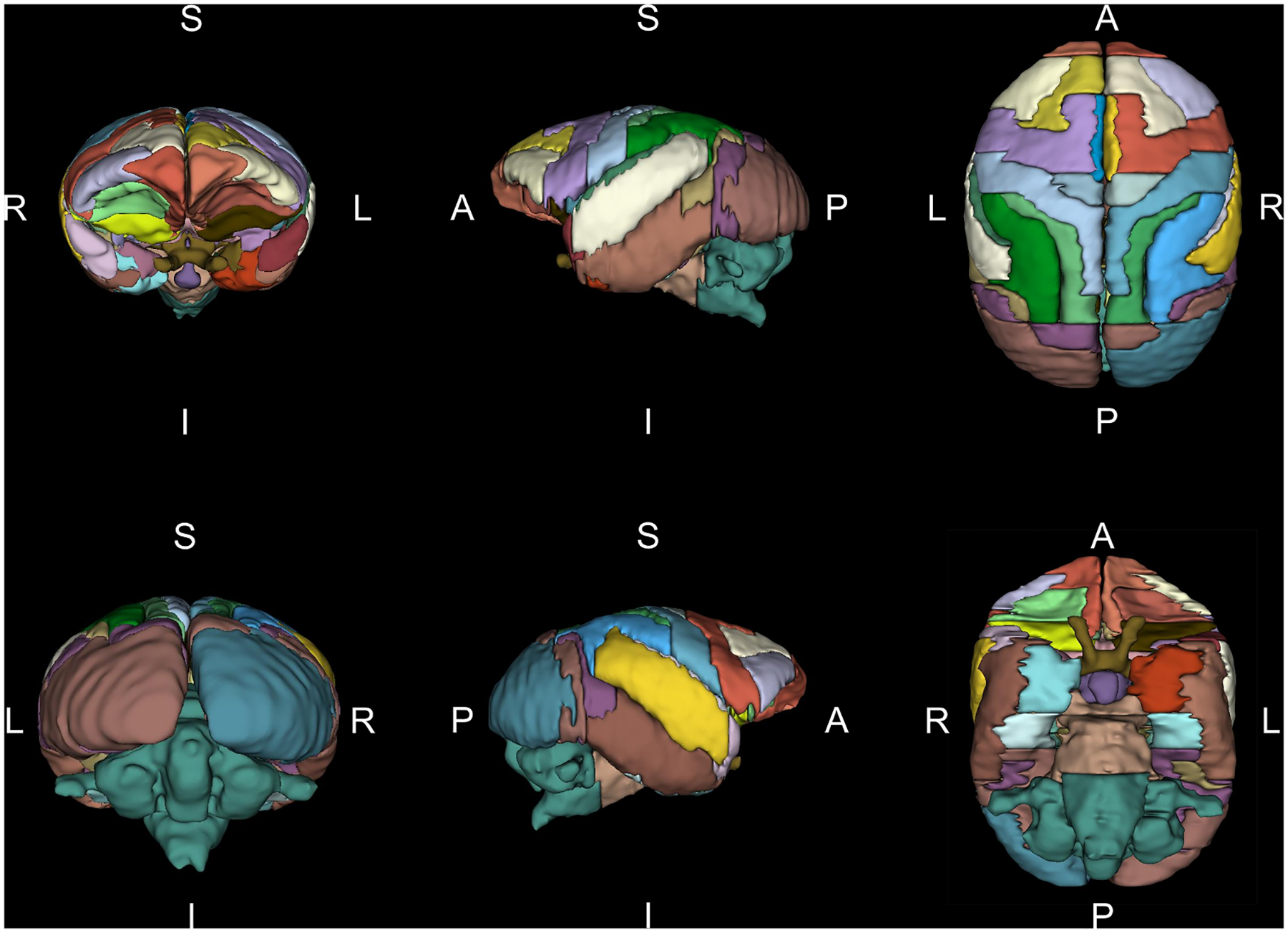
3D renderings of hierarchical Level 3 of UCD-TiNA shown from six observer perspectives: anterior (row 1, column 1), posterior (row 2, column 1), left (row 1, column 2), right (row 2, column 2) superior (row 1, column 3) and inferior (row 3, column 3).

Three-dimensional renderings of VOIs from Level 3 are illustrated in Fig. 5. The total brain volume was 22.90 cm^3^, comprising gray matter (13.73 cm^3^; cortical 8.52 cm^3^, subcortical 5.21 cm^3^) and white matter (3.33 cm^3^), noting that white matter volume is likely underestimated, as small tracts could not be reliably segmented. Comparative analyses showed that titi brains are 3.8-fold smaller than those of adult rhesus monkeys (Wisco et al., 2008) and 52.3-fold smaller than adult humans (Ge et al., 2002) in gray matter volume; and 7.9-fold smaller than rhesus and 150.1-fold smaller than human brains in white matter volume. The cerebellar volume of the titi monkey was 4.7-fold lower than that of the adult rhesus macaques. The gray-to-white matter ratio (4.12) exceeded that of both adult rhesus (1.81) and the adult human brains (1.2–1.5) (Wisco et al., 2008; Ge et al., 2002), reflecting a higher cortical density relative to white matter in the titi monkey.

### 3.2. Consistent overlap in VOIs generated through warping method compared to manual segmentation

The median DC and HD comparing regional VOIs from UCD-TiNA_mac to the those generated by the warping algorithm are presented in Table 3. Higher DC values were observed in cortical VOIs such as the inferior temporal cortex (0.831) and superior temporal region (0.825) as well as subcortical VOIs including the caudate (0.827) and putamen (0.806), which also exhibited lower HD (caudate: 1.121 mm and putamen: 1.470 mm). The thalamic VOIs showed moderate overlap (median DC: 0.763) and relatively low HD (median HD: 1.590). In contrast, the rhinal cortex demonstrated poorer performance with both lowest DC (0.595) and highest HD (4.045), likely due to its placement in the ventral region of the brain, where partial volume effects and proximity to cerebrospinal fluid boundaries may reduce segmentation fidelity. Intra-rater comparison yielded a median DC of 0.70 (range: 0.63–0.79) and median HD of 2.03 (range: 0.89–3.50) Inter-rater comparison between the expert and UCD-TiNA yielded a median DC of 0.76 (range: 0.63–0.82) and median HD of 1.62 (range: 0.97–2.51). Comparison between the primary segmenter and the expert yielded a median DC of 0.75 (range: 0.64–0.82) and median HD of 2.07 (range: 0.95–4.55). Supplementary Table 1 provides the DC and HD across regions. These results are comparable to published overlap metrics between trained human annotators for subcortical segmentation in other primate species (Tian et al., 2022; Fedorov et al., 2011; Ewert et al., 2019).

### 3.3. [^11^C]GR103545 BP_ND_ across brain VOIs delineated with UCD-TiNA

**Fig. 6.**
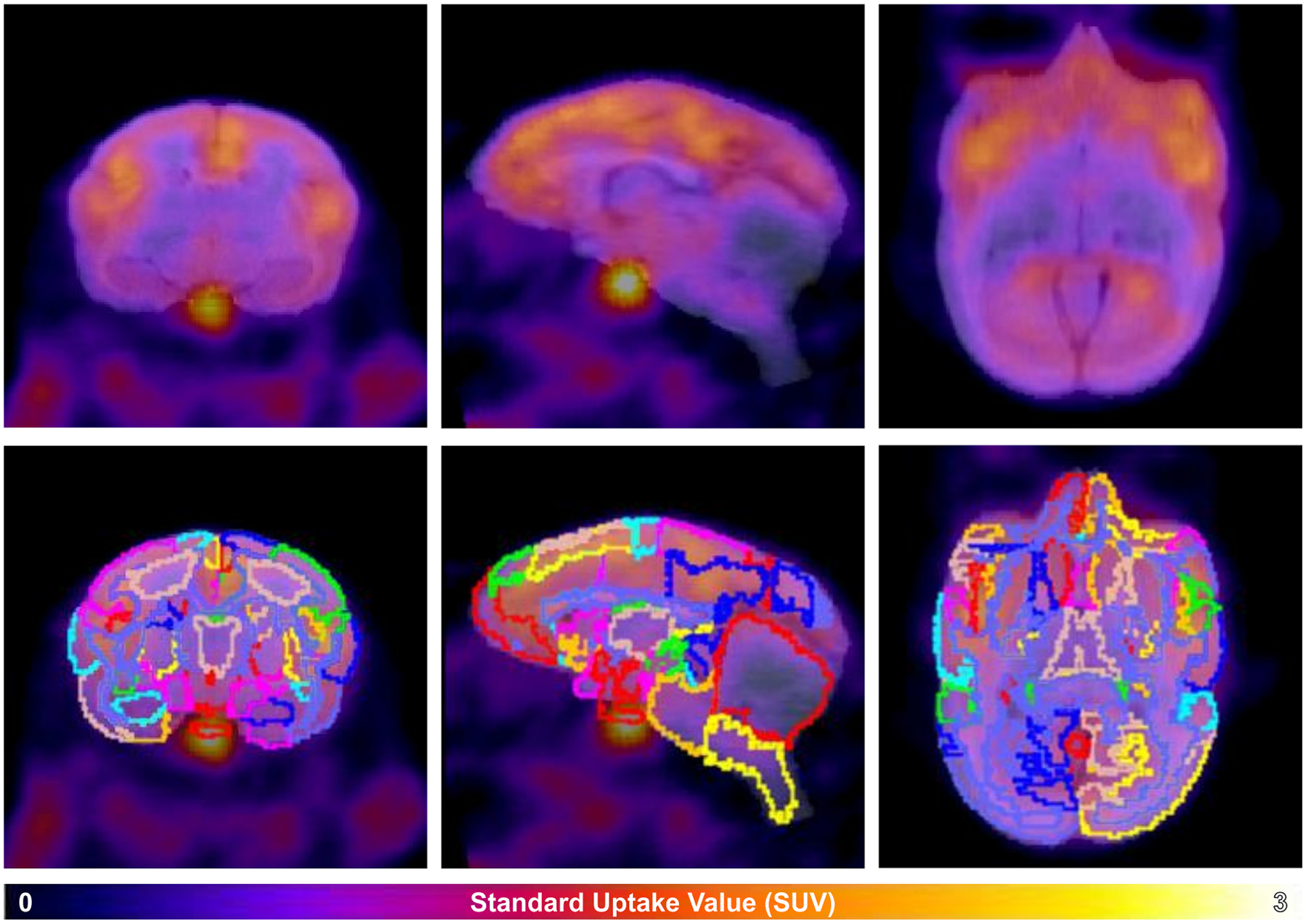
Coronal (column 1), sagittal (column 2) and axial (column 3) representations of [^11^C]GR103545 uptake in the titi monkey brain (row 1). The PET data (in color) overlaid on the MRI (grayscale). UCD-TiNA Level 3 VOI outlines are overlaid on the PET data in row 2.

Regional uptake patterns for [^11^C]GR103545 across the titi monkey brain along with an overlay of UCD-TiNA delineations are shown in Fig. 6 and average BP_ND_ for the 14 VOIs are reported in Table 4. Radiotracer uptake reproduced expected KOR density patterns reported in prior studies (Almeida et al., 2024); BP_ND_ was highest in the insula (BP_ND_ = 1.73), a dense KOR region, with moderate uptake in temporal VOIs (inferior temporal cortex BP_ND_ = 0.85, superior temporal region BP_ND_ = 0.98), and low uptake in thalamic VOIs (ventral thalamus BP_ND_ = 0.43, medial thalamus BP_ND_ = 0.45).

### 3.4. Minor differences in BP_ND_ when comparing manually-segmented VOIs to those generated by warping

**Fig. 7.**
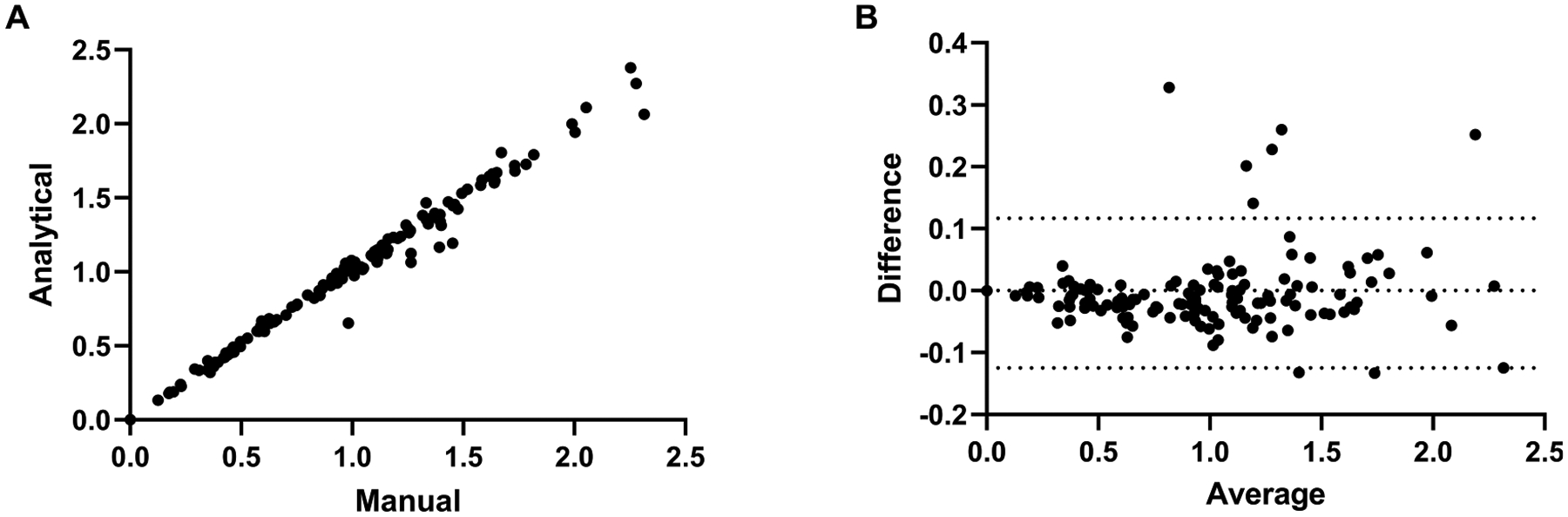
BP_ND_ differences between VOIs drawn manually and those obtained through the SyN warping of UCD-TiNA. (A) Correlation in BP_ND_ between the two methods (r = 0.992, p < 0.001). (B) Bland Altman plot comparing the difference versus the average BP_ND_, the solid line indicates the mean and the dotted lines the 95% CI.

We compared BP_ND_ values derived from manually-segmented UCD-TiNA_mac VOIs (N = 6 animals, 14 VOIs per animal) to corresponding estimates from ANTs B-Spline SyN warping of UCD-TiNA. Across all 84 VOI-animal pairs, median percentage difference was 0.63% (Table 5), with differences approaching 5% in two VOIs: somatosensory cortex (−4.39%) and inferior temporal cortex (−4.93%), both likely reflecting minor atlas-to-subject anatomical variation. Fig. 7 shows the results of correlation and Bland-Altman analysis. The Spearman correlation was r = 0.992 (p < 0.001), mean bias was −0.004 with [−0.124,0.116] as the 95% limits of agreement, and RMSE was 0.075. In a few temporal VOIs, such as the rhinal cortex, model fitting in 2/6 animals was unreliable, due to partial voluming with the high-binding pituitary gland.

## 4. Discussion

This work introduces a comprehensive atlas package for titi monkey neuroimaging that mitigates manual segmentation bottlenecks and establishes a whole-brain anatomic reference for standardized, reproducible image analysis workflows in AC-PC space. The primary atlas, UCD-TiNA, comprises 74 hierarchically-organized brain VOIs, manually segmented by trained researchers and experts. The three-level hierarchy provides flexibility across image resolutions, while separate segmentation of the left and right hemispheres avoids errors in delineations that assume bilateral symmetry.

The second component of atlas package, UCD-TiNA_group, provides a population-based atlas that includes animals across a range of adult ages across both male and female monkeys. While single-subject atlases often yield the most accurate segmentations for individuals with high structural similarity to the reference (Seidlitz et al., 2018), they may unevenly introduce bias when analyzing populations of individuals with naturally-occurring anatomic variation. To address this, the age range for UCD-TiNA_group was selected to mitigate the potential for age-related atrophy while hopefully capturing variability within the sexes during adulthood. This design should offer a neutral, generalizable starting-point for initializing warping algorithms, reducing the number of deformations needed, thereby minimizing error in these algorithms and improving the robustness of the analysis pipeline. An additional advantage of UCD-TiNA_group is that all monkeys were scanned on the same scanner with identical protocols, ensuring that observed anatomical variations reflect true biological differences rather than technical artifacts, a key advantage for registration accuracy.

Our third component, UCD-TiNA_mac, consists of a 14-VOI manually-segmented compilation of single-subject segmentations, focusing on brain VOIs implicated in social bonding – the primary application for this animal model. This compilation should enable multi-atlas segmentation (Lötjönen et al., 2010; Artaechevarria et al., 2009), which have recently gained traction, particularly with advances in neural network-based approaches (Cao et al., 2012; Manjón et al., 2018; Termenon et al., 2016). The hand-curated single-subject segmentations within UCD-TiNA_mac can therefore serve as a robust reference resource for benchmarking the performance of both analytical and machine learning based segmentation methods. Our validation demonstrated high concordance between manual and warped segmentations confirming the utility of UCD-TiNA_mac.

Among atlases in rhesus macaques, the surrogate D99 (Reveley et al., 2016), created by warping the original D99 (McLaren et al., 2009) (an MRI atlas derived from corresponding histological sections of the same monkey) to a high-resolution *ex vivo* scan of a male rhesus monkey at 0.25 mm^3^ isotropic voxel size in AC-PC space, remains the widely used reference. More recently, the NMT v2 template (Jung et al., 2021) was introduced with an isotropic voxel size of 0.25 mm^3^, created in Horsley-Clark stereotaxic orientation with two hierarchical atlases, CHARM (Jung et al., 2021) and SARM (Hartig et al., 2021). The CHARM and SARM atlases were chosen as our reference due to their 5-level hierarchical structure, anatomical homology, and provision for anatomical flexibility across image resolutions. Unlike other NHPs, histological atlases available for the titi monkey are limited. Therefore, our method employed a hybrid approach of comparative neuroanatomical methods alongside available histological and autoradiography data to create an anatomically-grounded parcellation scheme, leveraging gray-white matter contrast and landmarks recognizable across primates. Furthermore, we adopted the AC-PC orientation, the foundational stereotaxic framework across species, including marmosets, rather than Horsley-Clark coordinates (Jensen et al., 1996), which are designed for invasive procedures (surgery, electrophysiology, drug infusions) not commonly employed in social neuroscience experimental paradigms typical for the titi monkey. Lastly, adapting the atlas from a more anatomically complex NHP, the rhesus macaque, rather than extrapolating from a less complex NHPs, allowed us to leverage a more comprehensive set of VOIs and thereby improve anatomical fidelity.

Among published NHP atlases for the common marmoset (*Callithrix jacchus*), the atlas by Newman et al. (2009) uses digitized histologic images and high-resolution *ex vivo* MRI scans of a female marmoset. This atlas overlaid regional labels on native MRI rather than delineating them, limiting its utility as a standardized, anatomical reference. A more recent MRI-based hierarchical marmoset brain atlas (Liu et al., 2018) was created in AC-PC space via manual segmentation, guided by multi-modal *ex vivo* MRI data (magnetization transfer, T_2_-weighted, diffusion with fractional anisotropy) using a male marmoset monkey, with refinement via probabilistic tracking of cortical anisotropy. *Ex vivo* atlases, despite not being limited by physiological noise or anesthesia duration, introduce systemic limitations. Chemical fixation and post-mortem processing alter brain volume, water content, and tissue magnetic properties (hence, contrast) (Iglesias et al., 2015), causing non-rigid distortions (Jung et al., 2021; Weiss et al., 2021; Holmes et al., 2017), such as ventricle shrinkage, sulcal banks compression, and collapse of cerebrospinal fluid spaces (Holmes et al., 2017; Santini et al., 2025; Seifert et al., 2019). While *ex vivo* limitations motivated our *in vivo* approach to atlas creation, incorporating multi-contrast, high-resolution imaging, as successfully applied in the marmoset atlas, is a logical next step to refine subcortical and cortical boundaries of our atlas.

Our validation of UCD-TiNA using PET demonstrated that [^11^C] GR103545 binding patterns reproduced expected KOR distributions (Almeida et al., 2024) and correlated highly with manual segmentation (r = 0.992, median BP_ND_ difference <1%). The limitation in ventral temporal VOIs situated near the pituitary reflects a local partial-volume challenge inherent to animal imaging at this resolution, not an atlas-specific deficiency. This limitation, as such, affected both manual and automated segmentation equally and users should apply *post-hoc* anatomical refinement or interpret quantification in these regions with caution.

*In vivo* imaging of a non-human primate species with an average weight of 1.3 kg on a 1.5T scanner with 1 mm slice thickness inevitably introduced partial voluming and limits the achievable contrast- and signal-to-noise ratio. Although our manual segmentation approach attenuated some concern, our ability to delineate finer structures and segment deeper anatomical hierarchies was limited. Furthermore, all segmentations of the UCD-TiNA compilation were performed by a single user. Although intra- and inter-rater variability were systematically evaluated and were comparable to those reported in the published literature (Tian et al., 2022; Fedorov et al., 2011; Ewert et al., 2019), broader validation across independent users would strengthen confidence in the atlas’s generalizability. This atlas therefore should be regarded as a first-generation resource that will benefit from future higher-field, higher-resolution acquisitions.

Accordingly, planned enhancements to our atlas package involve scanning titi monkeys at higher field strengths with increased resolution, and therefore deeper hierarchical subdivisions, comparison of high-resolution *ex vivo* brain scans with the current atlas, to potentially enable more precise delineations of subcortical structures, and inclusion of larger subsets of manual segmentations into UCD-TiNA_mac, to better characterize anatomical variability. To further enhance the generalizability of UCD-TiNA_group, developing age-specific templates derived from larger cohorts and explicit age-stratified analyses will be important. These improvements would enable further optimization of the warping pipeline. Lastly, establishing standardized guidelines for manual corrections in regions prone to lower Dice coefficients would further improve the applicability of this work. These developments would expand atlas utility for investigating not only pair-bonding circuits but also broader dimensions of social behavior, stress responsivity, and psychiatric disease models with direct relevance to human attachment disorders.

In conclusion our MRI brain atlas package mitigates key technical barriers to quantitative, region-specific neuroimaging in the titi monkey, by enabling automated, unbiased analyses beyond the limited set of regions feasible with manual segmentation. This should aid in employing structural morphometry, functional imaging and receptor PET to interrogate the neural circuitry of pair bonding, attachment behavior and social connectedness. Open access to the atlas package and pipelines will support reproducible, multi-center studies, accelerating efforts to link specific brain circuits to variation in social behavior and to translational models of human attachment and related psychiatric disorders.

## Supplementary Material

Supplementary materials

Supplementary material associated with this article can be found, in the online version, at doi:10.1016/j.neuroimage.2026.121921.

## Figures and Tables

**Table 1:** Distribution of VOIs across the three hierarchical levels of UCD-TiNA in cortical and subcortical gray matter as well as white matter. From left to right, cells shaded in blue and purple indicate cortical and subcortical VOIs correspondingly. Level 2 VOIs are shaded in yellow while Level 3 VOIs are shaded in green. Note that Level 2 VOIs that were not further subdivided at Level 3 remain shaded in green, indicating that they represent the highest hierarchical level for those VOIs.

	Level 1	Level 2	Bilateral	Abbreviation	Level 3	Bilateral	Abbreviation
Cortical	Frontal Lobe	Anterior Cingulate Gyrus	Yes		Anterior Cingulate Cortex	Yes	ACC
Cortical	Frontal Lobe	Anterior Cingulate Gyrus	Yes		Mid Cingulate Cortex	Yes	MCC
Cortical	Frontal Lobe	Orbitofrontal Cortex	Yes		Medial Orbitofrontal Cortex	Yes	med_OFC
Cortical	Frontal Lobe	Orbitofrontal Cortex	Yes		Lateral Orbitofrontal Cortex	Yes	lat_OFC
Cortical	Frontal Lobe	Orbitofrontal Cortex	Yes		Caudal Orbitofrontal Cortex	Yes	caudal_OFC
Cortical	Frontal Lobe	Lateral Prefrontal Cortex	Yes		Dorsolateral Prefrontal Cortex	Yes	dIPFC
Cortical	Frontal Lobe	Lateral Prefrontal Cortex	Yes		Ventrolateral Prefrontal Cortex	Yes	vIPFC
Cortical	Frontal Lobe	Motor Cortex	Yes		Medial Supplementary Motor Areas	Yes	SMA
Cortical	Frontal Lobe	Motor Cortex	Yes		Premotor Cortex	Yes	PM
Cortical	Frontal Lobe	Motor Cortex	Yes		Primary Motor Cortex	Yes	M1
Cortical	Parietal Lobe	Somatosensory Cortex	Yes	SSC	-	-	-
Cortical	Parietal Lobe	Superior Parietal Lobule	Yes	SPL	-	-	-
Cortical	Parietal Lobe	Inferior Parietal Lobule	Yes	IPL	-	-	-
Cortical	Parietal Lobe	Posterior Medial Cortex	Yes	PMC	-	-	-
Cortical	Temporal Lobe	Medial Temporal Lobe	Yes		Parahippocampal Cortex	Yes	paraHipp
Cortical	Temporal Lobe	Medial Temporal Lobe	Yes		Rhinal Cortex	Yes	RC
Cortical	Temporal Lobe	Temporal Pole	Yes	TG	-	Yes	
Cortical	Temporal Lobe	Inferior Temporal Cortex	Yes	ITC	-	Yes	
Cortical	Temporal Lobe	Superior Temporal Cortex	Yes		Superior Temporal Region	Yes	STG
Cortical	Temporal Lobe	Superior Temporal Cortex	Yes		Core and Belt Areas of Auditory Cortex	Yes	core/belt
Cortical	Temporal Lobe	Insula	Yes	Ins	-	-	-
Cortical	Occipital Lobe	Primary Visual Cortex	Yes	V1	-	-	-
Cortical	Occipital Lobe	Extrastriate Visual Areas	Yes		V2-V3	Yes	V2-V3
Cortical	Occipital Lobe	Extrastriate Visual Areas	Yes		V4	Yes	V4
Subcortical	Telencephalon	Lateral and Ventral Pallium	Yes		Claustrum	Yes	CI
Subcortical	Telencephalon	Lateral and Ventral Pallium	Yes		Ventral Pallium	Yes	Vpal
Subcortical	Telencephalon	Lateral and Ventral Pallium	Yes		Piriform Cortex	Yes	Pir
Subcortical	Telencephalon	Medial Pallium	No		Hippocampus	Yes	Hi
Subcortical	Telencephalon	Medial Pallium	No		Fornix	No	f
Subcortical	Telencephalon	Diagonal Subpallium	No		Lateral Septum	No	LS
Subcortical	Telencephalon	Diagonal Subpallium	No		Basal Nucleus Region	No	BR
Subcortical	Telencephalon	Pallidum	No		Globus Pallidus	Yes	GP
Subcortical	Telencephalon	Pallidum	No		Anterior Commisure	No	ac
Subcortical	Telencephalon	Pallidum	No		Ventral Pallidum	Yes	VP
Subcortical	Telencephalon	Amygdala	Yes	Amy	-	-	-
Subcortical	Telencephalon	Preoptic_Complex	No	POC	-	-	-
Subcortical	Telencephalon	Striatum	Yes		Caudate	Yes	Cd
Subcortical	Telencephalon	Striatum	Yes		Putamen	Yes	Pu
Subcortical	Telencephalon	Striatum	Yes		Internal Capsule	Yes	ic
Subcortical	Telencephalon	Striatum	Yes		Amygdalostriatal_Transition	Yes	Ast
Subcortical	Telencephalon	Striatum	Yes		Nucleus Accumbens	Yes	NAc
Subcortical	Diencephalon	Hypothalamus	No	Hy	-	-	-
Subcortical	Diencephalon	Epithalamus	No	EpiThal	-	-	-
Subcortical	Diencephalon	Thalamus	No		Ventral Thalamus	No	Vthal
Subcortical	Diencephalon	Thalamus	No		Medial Thalamus	No	Mthal
Subcortical	Mesencephalon	Midbrain	No		Dorsal Midbrain	No	Dmid
Subcortical	Mesencephalon	Midbrain	No		Lateral Midbrain	No	Lmid
Subcortical	Mesencephalon	Midbrain	No		Medial Midbrain	No	Mmid
Subcortical	Mesencephalon	Midbrain	No		Ventral Midbrain	No	Vmid
Subcortical	Metencephalon	Pons	No	pons	-	-	-
Subcortical	Metencephalon	Cerebellum	No	Cb	-	-	-
Subcortical	Myelencephalon	Medulla	No	Med	-	-	-
White Matter		White matter tracts	Yes		White matter tracts	No	
White Matter		White matter tracts	Yes		Centrum Semiovale	No	CSO

**Table 2:** VOI statistics for hierarchical Level 3 of UCD-TiNA, including voxel count, label map volume in cm^3^ (calculated as voxel count x voxel volume), VOI surface area in cm^2^ and VOI volume derived from the closed surface model in cm^3^.

Index	Segment	Voxel count	Label map volume [10^−3^ cm^3^]	Surface area [10^−2^ cm^2^]	Volume [10^−3^ cm^3^]
1	Ant_Cingulate_Cortex_L	3159	101.80	162.98	98.52
2	Ant_Cingulate_Cortex_R	3203	103.22	166.01	99.63
3	Mid_Cingulate_Cortex_L	2816	90.75	119.89	89.20
4	Mid_Cingulate_Cortex_R	2662	85.79	117.70	83.98
5	Medial_Orbitofrontal_Cortex_L	3690	118.92	191.67	114.61
6	Medial_Orbitofrontal_Cortex_R	3752	120.91	196.17	115.92
7	Lateral_Orbitofrontal_Cortex_L	2916	93.97	168.77	90.38
8	Lateral_Orbitofrontal_Cortex_R	2678	86.30	158.65	82.99
9	Caudal_Orbitofrontal_Cortex_L	2605	83.95	170.77	80.53
10	Caudal_Orbitofrontal_Cortex_R	2413	77.76	156.97	75.06
11	Dorsolateral_Prefrontal_Cortex_L	4215	135.84	189.42	132.83
12	Dorsolateral_Prefrontal_Cortex_R	3224	103.90	168.81	100.84
13	Ventrolateral_Prefrontal_Cortex_L	4418	142.38	188.53	139.84
14	Ventrolateral_Prefrontal_Cortex_R	3993	128.68	182.11	125.97
15	Medial_Supplementary_Motor_Areas_L	1886	60.78	104.25	58.73
16	Medial_Supplementary_Motor_Areas_R	1620	52.21	96.48	50.23
17	Premotor_Cortex_L	7254	233.77	330.92	229.40
18	Premotor_Cortex_R	7751	249.79	359.50	245.74
19	Primary_Motor_Cortex_L	1448	46.66	76.74	45.15
20	Primary_Motor_Cortex_R	1266	40.80	72.37	38.99
21	Somatosensory Cortex_L	8571	276.21	405.71	270.97
22	Somatosensory Cortex_R	8360	269.41	403.01	264.31
23	Superior_Parietal_Lobule_L	5806	187.11	310.35	182.57
24	Superior_Parietal_Lobule_R	5435	175.15	288.84	169.85
25	Inferior_Parietal_Lobule_L	8841	284.92	386.38	280.94
26	Inferior_Parietal_Lobule_R	9960	320.98	429.46	316.52
27	Posterior_Medial_Cortex_L	3411	109.93	177.06	106.61
28	Posterior_Medial_Cortex_R	3760	121.17	187.67	117.15
29	Parahippocampal_Cortex_L	1562	50.34	113.58	46.32
30	Parahippocampal_cortex_R	1798	57.94	117.64	54.20
31	Rhinal_Cortex_L	3780	121.82	190.46	117.52
32	Rhinal_Cortex_R	4181	134.74	216.31	130.35
33	Temporal_Pole_L	2006	64.65	136.17	61.43
34	Temporal_Pole_R	1227	39.54	94.44	35.89
35	Inferior_Temporal_Cortex_L	11,152	359.39	458.02	352.77
36	Inferior_Temporal_Cortex_R	11,841	381.60	469.06	375.64
37	Superior_Temporal_Region_L	10,333	333.00	420.77	328.73
38	Superior_Temporal_Region_R	9744	314.02	394.56	307.95
39	CABA_L	3500	112.79	213.53	106.06
40	CABA_R	3621	116.69	197.47	112.03
41	Insula_L	2931	94.46	160.20	90.96
42	Insula_R	2309	74.41	139.51	70.01
43	V1_L	21,238	684.43	924.51	676.73
44	V1_R	20,975	675.95	950.26	667.58
45	V2V3_L	14,997	483.30	839.99	466.80
46	V2V3_R	16,401	528.55	879.86	514.33
47	V4_L	3313	106.77	206.76	100.59
48	V4_R	3217	103.67	207.72	98.48
49	Claustrum_L	2119	68.29	142.39	61.47
50	Claustrum_R	1829	58.94	116.36	52.57
51	Ventral_Pallium_L	622	20.04	55.42	15.92
52	Ventral_Pallium_R	785	25.30	64.16	22.12
53	Piriform_Cortex_L	230	7.41	20.74	6.06
54	Piriform_Cortex_R	402	12.96	35.01	11.16
55	Hippocampus_L	4086	131.68	177.15	129.16
56	Hippocampus_R	4057	130.74	179.83	127.99
57	Fornix	738	23.78	70.17	15.49
58	Lateral_Septum	1908	61.49	115.07	56.01
59	Basal_Nucleus_Region	1442	46.47	105.92	41.74
60	Globus_Pallidus_L	3494	112.60	142.52	110.31
61	Globus_Pallidus_R	3340	107.64	149.52	104.45
62	Anterior_Commissure	829	26.72	73.40	20.80
63	Ventral_Pallidum_L	226	7.28	20.23	5.02
64	Ventral_Pallidum_R	267	8.60	23.62	6.34
65	Amygdala_L	2644	85.21	113.55	83.83
66	Amygdala_R	2528	81.47	109.51	79.86
67	Preoptic_Complex	3444	110.99	228.60	101.70
68	Caudate_L	4276	137.80	214.84	132.27
69	Caudate_R	4235	136.48	217.85	130.08
70	Putamen_L	5143	165.74	203.41	161.21
71	Putamen_R	4588	147.86	186.82	144.34
72	Internal_Capsule_L	2271	73.19	139.71	68.62
73	Internal_Capsule_R	2266	73.03	135.86	67.91
74	Amygdalostriatal_Transition_L	728	23.46	50.90	21.00
75	Amygdalostriatal_Transition_R	802	25.85	48.25	23.52
76	Nucleus_Accumbens_L	316	10.18	23.69	9.03
77	Nucleus_Accumbens_R	284	9.15	21.99	8.18
78	Hypothalamus	4858	156.56	226.95	153.16
79	Epithalamus	597	19.24	52.52	15.29
80	Ventral_Thalamus	10,348	333.48	529.92	320.62
81	Medial_Thalamus	4610	148.56	192.30	143.84
82	Dorsal_Midbrain	5475	176.44	220.60	173.13
83	Lateral_Midbrain	3616	116.53	212.37	110.88
84	Medial_Midbrain	573	18.47	44.29	15.65
85	Ventral_Midbrain	4787	154.27	224.95	149.49
86	Pons	13,275	427.81	383.34	422.35
87	Cerebellum	48,811	1573.01	917.25	1567.34
88	Medulla	10,061	324.23	290.53	321.45
90	Centrum_Semiovale	5413	174.44	249.80	170.01

**Table 3:** Median Dice coefficients, a metric of overlap, and Hausdorff distance, a metric to determine the extent of deviation from the region’s boundary (in mm) for the 14 VOIs from our manually-segmented atlas compilation, UCD-TiNA_mac, with N = 6 animals comparing manual segmentations to those generated using ANTs B-Spline SyN.

VOI	Dice coefficient	Hausdorff Distance (mm)
Dorsolateral Prefrontal Cortex	0.766	1.888
Somatosensory Cortex	0.734	3.140
Rhinal Cortex	0.595	4.045
Inferior Temporal Cortex	0.831	2.456
Superior Temporal Region	0.825	2.290
Insula	0.751	1.656
Septum	0.736	2.034
Amygdala	0.675	2.177
Caudate	0.827	1.121
Putamen	0.806	1.470
Nucleus Accumbens	0.659	1.007
Hypothalamus	0.763	1.590
Ventral Thalamus	0.681	1.842
Medial Thalamus	0.765	1.174

**Table 4:** BP_ND_ (mean [95% CI]) for N = 42 [^11^C]GR103545 PET scans in 14 VOIs delineated by warping UCD-TiNA hierarchical Level 3 to each animal’s individual MRI.

VOI	BP_ND_ (mean and 95% CI)
Dorsolateral Prefrontal Cortex	1.20 [1.08–1.32]
Somatosensory Cortex	1.20 [1.10–1.31]
Rhinal Cortex	1.01 [0.93–1.09]
Inferior Temporal Cortex	0.85 [0.78–0.91]
Superior Temporal Region	0.98 [0.90–1.06]
Insula	1.73 [1.63–1.84]
Septum	1.19 [1.11–1.27]
Amygdala	1.28 [1.19–1.38]
Caudate	1.07 [1.00–1.14]
Putamen	1.43 [1.34–1.53]
Nucleus Accumbens	1.63 [1.51–1.75]
Hypothalamus	1.15 [1.06–1.24]
Ventral Thalamus	0.43 [0.40–0.45]
Medial Thalamus	0.45 [0.42–0.47]

**Table 5:** Median BP_ND_’s reported in N = 6 [^11^C]GR103545 PET scans with the 14 VOIs derived from UCD-TiNA_mac (column 2), as well as ANTs SyN warping of UCD-TiNA to each of the same animals (column 3). Percentage change in BP_ND_ values (column 4) between the two methods.

VOI	BP_ND_ (manual)	BP_ND_ (ANTs_SyN)	Percentage change (%)
Dorsolateral Prefrontal Cortex	1.06	1.07	0.34
Somatosensory Cortex	0.90	0.91	−4.39
Rhinal Cortex	1.01	0.95	2.01
Inferior Temporal Cortex	0.72	0.75	−4.93
Superior Temporal Region	0.83	0.85	−1.85
Insula	1.45	1.48	1.05
Septum	1.11	1.13	−2.90
Amygdala	1.10	1.11	0.06
Caudate	0.95	0.98	−1.57
Putamen	1.37	1.38	−1.42
Nucleus Accumbens	1.71	1.67	0.28
Hypothalamus	0.96	0.97	−1.32
Ventral Thalamus	0.38	0.37	1.80
Medial Thalamus	0.42	0.42	0.55
