# Supplementary material for: A hierarchical brain MRI atlas of the coppery titi monkey (Plecturocebus cupreus)
Source: Neuroimage. Author manuscript; Available in PMC 2026 May 21. (PMC13193678; doi:10.1016/j.neuroimage.2026.121921)
Supplement: Supplementary materials [file NIHMS2173105-supplement-Supplementary_materials.docx]

**Supplementary Material**

**Title:** A Hierarchical Brain MRI Atlas of the Coppery Titi Monkey (*Plecturocebus cupreus*)

Alita Jesal D Almeida^1,2^, Brad A. Hobson^3^, Anelise Caceres^1^, Sarah Tam^3^, John P. Paulus^4^, Claudia Manca^5^, Anand A. Joshi^6^, Sara M. Freeman^7^, Karen L. Bales^5,8*^ and Abhijit J. Chaudhari^2,3,8*^

^1^Department of Biomedical Engineering, University of California-Davis College of Engineering, Davis, CA, 95616, USA; ^2^Department of Radiology, University of California-Davis School of Medicine, Sacramento, CA, 95817, USA; ^3^Center for Molecular and Genomic Imaging, Department of Biomedical Engineering, University of California-Davis College of Engineering, Davis, CA, 95616, USA; ^4^Department of Neuroscience, University of California-Davis Center for Neuroscience, Davis, CA 95616, USA; ^5^Department of Psychology, University of California-Davis College of Letters and Science, Davis, CA 95616; ^6^Ming Hsieh Department of Electrical and Computer Engineering, University of Southern California, Los Angeles, CA 90089-2560; ^7^Department of Biology, Utah State University, Logan, UT 84322; ^8^California National Primate Research Center, Davis, CA 95616

***Corresponding authors**: Abhijit J. Chaudhari, Ph.D., Department of Radiology, University of California-Davis, 4860 Y Street, Suite 3100, Sacramento, CA 95817, Email: [ajchaudhari@ucdavis.edu](mailto:ajchaudhari@ucdavis.edu); Karen L. Bales, Ph.D., Department of Psychology, University of California-Davis, One Shields Ave, Davis, CA, 95616, USA, Email: [klbales@ucdavis.edu](mailto:klbales@ucdavis.edu)

**Figures:**


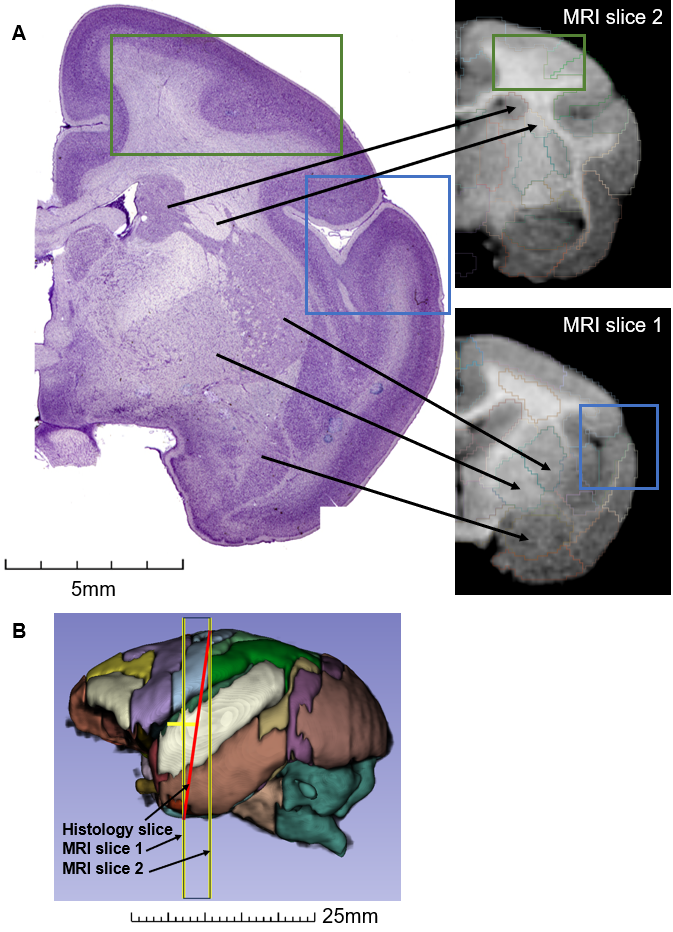


**Supplementary Figure 1:** Demonstration of VOI delineation approach with reference to a histological slice (thionin-stained) from the Cell Brain Atlas of the titi monkey^1^. (A) Correspondence between the anatomical structures is indicated by black arrows. Due to the slice angle of the histological data, alignment of structural landmarks is distributed across multiple MR images. Aligning cortical gyri and sulci (green and blue boxes) and white matter provided a reliable method for matching MRI slices with histological sections. (B) Histological sections are not in AC-PC space, therefore, the correspondence between the two MRI slices (yellow) and histological section (red) corresponding to (A) are highlighted on a 3D rendering of Level 3 VOIs to enable better visualization.


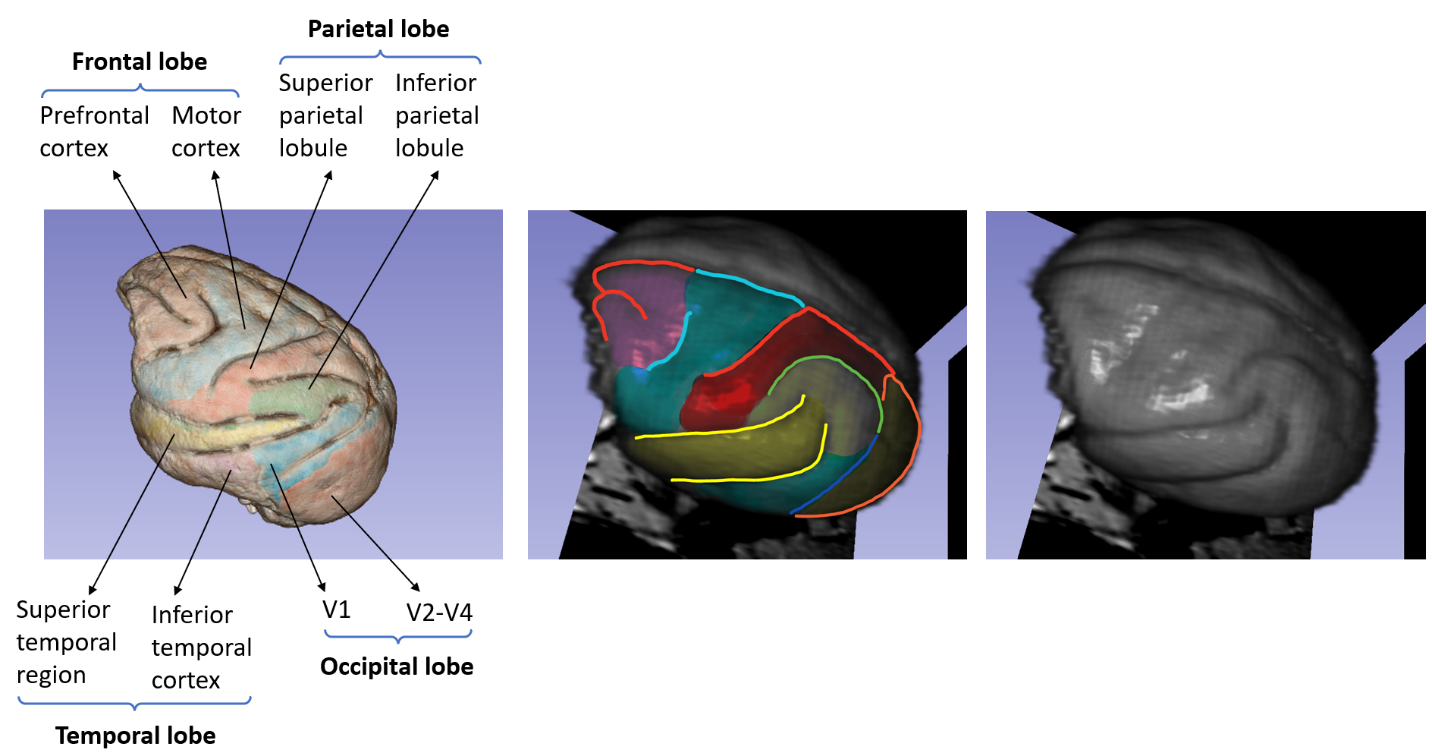


**Supplementary Figure 2:** Skull-stripped 3D renderings of rhesus brain overlaid with CHARM labels in color (left) and titi monkey brain (right) with select UCD-TiNA Level 2 VOIs (center) to illustrate lobular correspondence between species.

**
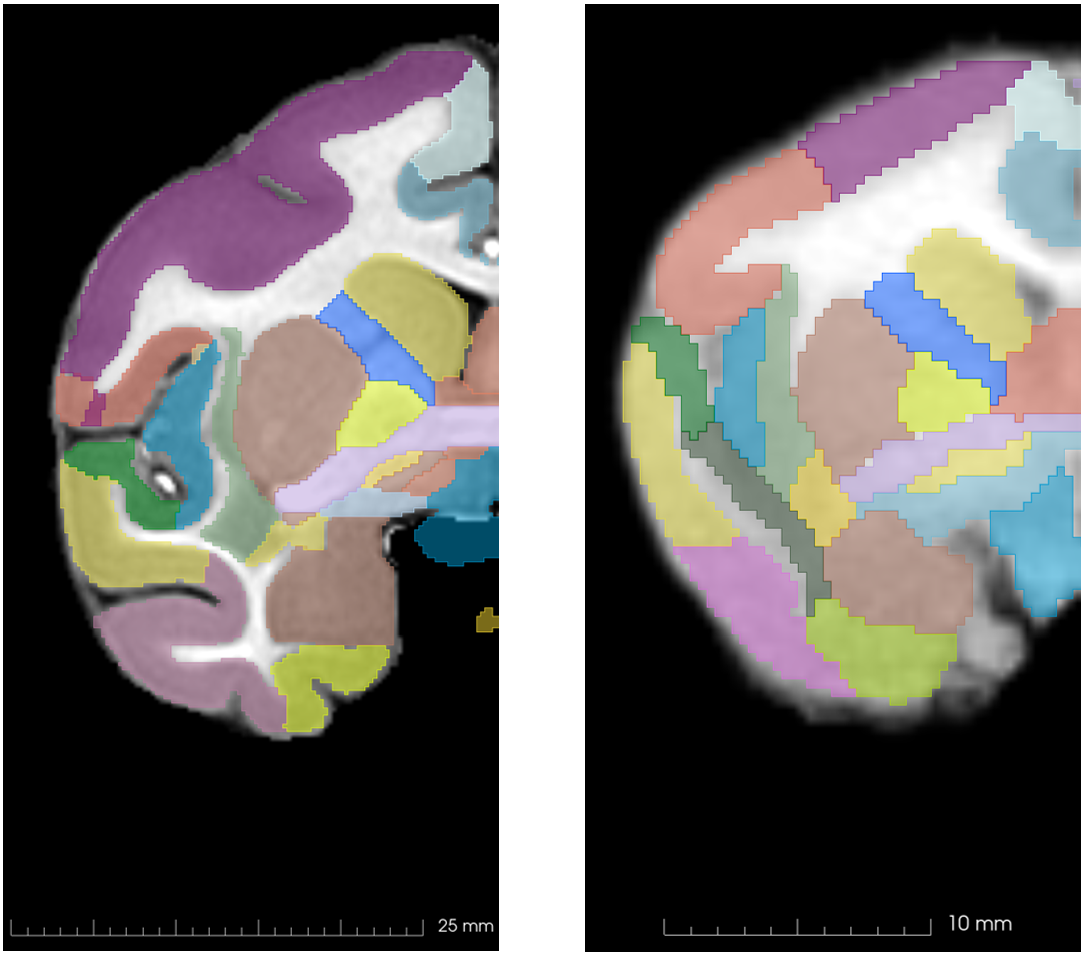
**

**Supplementary Figure 3:** Comparison of rhesus segmentations (left) with titi monkey segmentations (right). Corresponding regions are approximately color-matched. Although the sulci are less pronounced in the titi monkey, shallow indentations are present and served as landmarks to guide the cortical delineations.


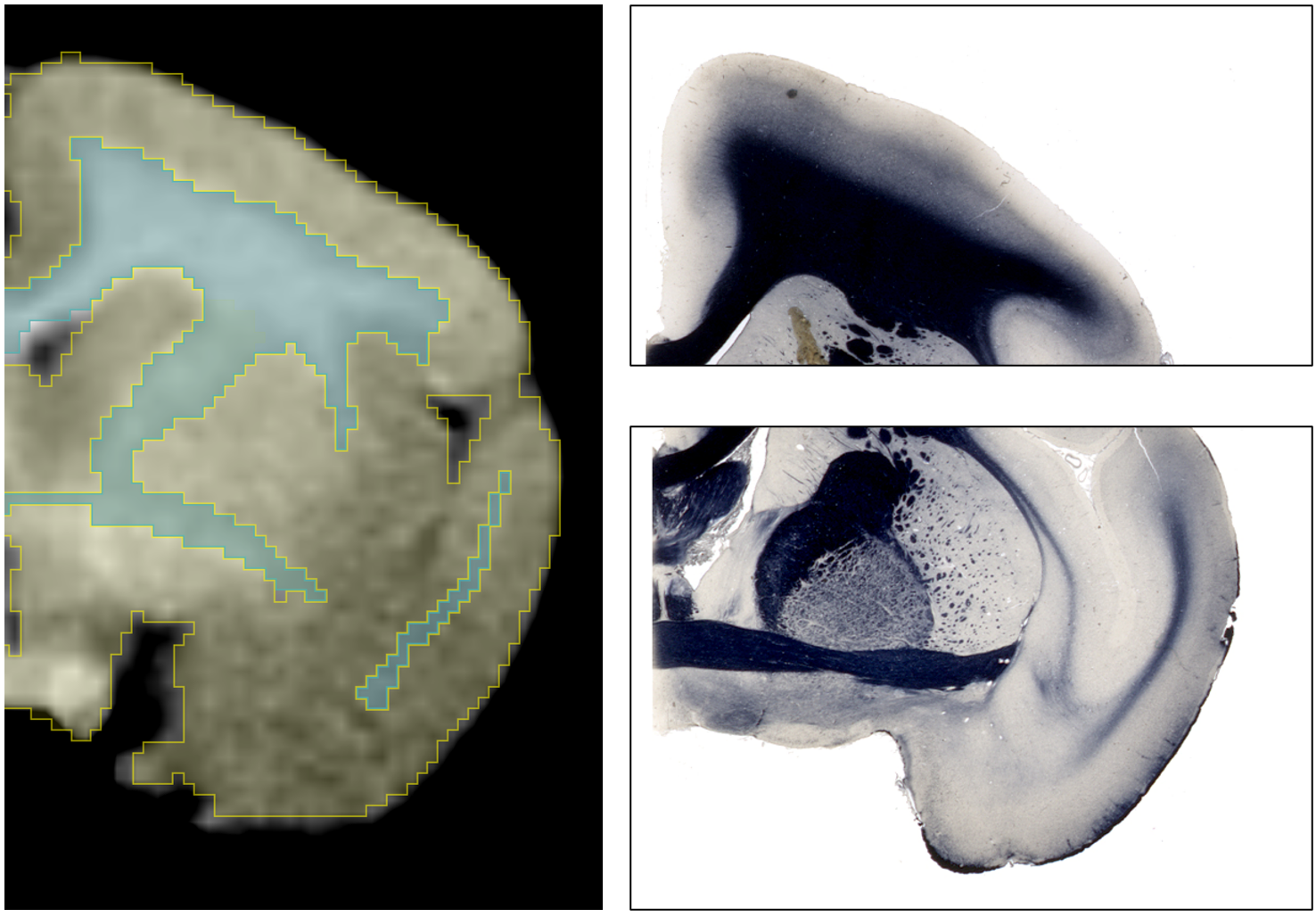


**Supplementary Figure 4:** Side-by-side comparison of manually-delineated white matter tracts (blue) from UCD-TiNA (left) and corresponding slices from the published hematoxylin-stained Fiber Brain Atlas for the titi monkey [1] (right) with matched white matter tracts. Although partial volume effects were present in the MRI, an overall homology of tract branching pattern is evident. As noted in Supplementary Figure 1, the slice orientation of the histological samples only approximates the AC-PC orientation used for UCD-TiNA, necessitating the use of multiple histological slices for accurate comparison with the MRI slice.

**Tables:**

| **Region Name** | **Dice coefficient** | | | **Hausdorff distance** | | |
| --- | --- | --- | --- | --- | --- | --- |
|  | **S1** | **S2** | **S1-S2** | **S1** | **S2** | **S1-S2** |
| Dorsolateral Prefrontal Cortex | 0.71 | 0.76 | 0.77 | 1.46 | 1.44 | 1.36 |
| Somatosensory Cortex | 0.67 | 0.70 | 0.74 | 2.48 | 1.98 | 2.14 |
| Rhinal Cortex | 0.63 | 0.74 | 0.64 | 2.87 | 2.10 | 3.31 |
| Inferior Temporal Cortex | 0.76 | 0.75 | 0.77 | 3.50 | 2.51 | 4.55 |
| Superior Temporal Region | 0.69 | 0.77 | 0.75 | 3.40 | 1.62 | 3.59 |
| Amygdala | 0.70 | 0.77 | 0.68 | 2.34 | 1.54 | 2.44 |
| Caudate | 0.76 | 0.82 | 0.76 | 1.27 | 1.20 | 2.07 |
| Putamen | 0.79 | 0.78 | 0.82 | 1.09 | 1.76 | 1.53 |
| Nucleus Accumbens | 0.64 | 0.63 | 0.69 | 0.89 | 0.97 | 0.95 |
| Ventral Thalamus | 0.69 | 0.73 | 0.73 | 2.03 | 1.72 | 1.65 |
| Medial Thalamus | 0.77 | 0.79 | 0.77 | 1.56 | 1.49 | 1.21 |

**Supplementary Table 1:** Dice coefficient (DC) and Hausdorff distance (HD) comparing delineations performed by the primary segmenter (S1) 8 months after initial segmentations and comparing the second segmenter’s (S2) delineations to UCD-TiNA’s delineations. The last column for each metric compares delineations between the S1 and the S2.

| **Method number** | **Method** | **Transform** | **Spline distance (Bspline)** | **Metric** | **HISTO matching** | **If HISTO matching, bin size** | **Gradient step size** | **Shrink** | **Convergence/ Number of iterations** |
| --- | --- | --- | --- | --- | --- | --- | --- | --- | --- |
| 1 | ANTs | SyN | - | CC | no | - | 0.1 | 3x2x1 | 1.00E-06 |
| 2 | ANTs | SyN | - | CC | no | - | 0.1 | 1x1x1 | 1.00E-06 |
| 3 | ANTs | SyN | - | CC | no | - | 0.1 | 3x2x1 | 1.00E-08 |
| 4 | ANTs | SyN | - | CC | yes | 32 | 0.1 | 3x2x1 | 1.00E-06 |
| 5 | ANTs | SyN | - | MI | no | - | 0.1 | 3x2x1 | 1.00E-06 |
| 6 | ANTs | SyN | - | MI | yes | 32 | 0.1 | 3x2x1 | 1.00E-06 |
| 7 | ANTs | SyN | - | MI | yes | 16 | 0.1 | 3x2x1 | 1.00E-06 |
| 8 | ANTs | Bspline SyN | 26 | CC | no | - | 0.1 | 3x2x1 | 1.00E-06 |
| 9 | ANTs | Bspline SyN | 26 | CC | no | - | 0.1 | 1x1x1 | 1.00E-06 |
| 10 | ANTs | Bspline SyN | 26 | CC | no | - | 0.1 | 3x2x1 | 1.00E-08 |
| 11 | ANTs | Bspline SyN | 39 | CC | no | - | 0.1 | 3x2x1 | 1.00E-06 |
| 12 | ANTs | Bspline SyN | 26 | MI | no | - | 0.1 | 3x2x1 | 1.00E-06 |
| 13 | ANTs | Bspline SyN | 16 | CC | no | - | 0.1 | 3x2x1 | 1.00E-06 |

**Supplementary Table 2:** Configurations implemented for optimization of the ANTs Symmetric Normalization (SyN) warping. Best performing configuration that was implemented in the manuscript is highlighted in green.

**References:**

[1] University of Wisconson-Madison Brain Collection. Comparative Mammalian Brain Collections: Titi Monkey (Callicebus moloch), https://brainmuseum.org/Specimens/primates/titimonk/index.html.
